# Spatial scanning for the identification of risk areas for fetal death: an ecological study in Pernambuco

**DOI:** 10.1590/1980-220X-REEUSP-2025-0248en

**Published:** 2026-01-09

**Authors:** Indianara Maria de Barros Canuto, Cristine Vieira do Bonfim, Amanda Priscila de Santana Cabral Silva

**Affiliations:** 1 Fundação Oswaldo Cruz, Instituto Aggeu Magalhães, Recife, PE, Brazil.; 2 Secretaria de Saúde do Recife, Distrito Sanitário II, Recife, PE, Brazil.; 3 Fundação Joaquim Nabuco, Recife, PE, Brazil.; 4 Universidade Federal de Pernambuco, Curso de Bacharelado em Saúde Coletiva, Vitória de Santo Antão, PE, Brazil.

**Keywords:** Fetal Mortality, Vital Statistics, Health Information Systems, Spatial Analysis, Epidemiological Monitoring

## Abstract

**Objective:**

To describe the epidemiological characteristics and the use of the spatial scanning technique to identify clusters of fetal deaths in Pernambuco, between 2013 and 2022.

**Method:**

An ecological study having the municipalities as the unit of analysis, carried out in Pernambuco. Data from the Mortality and Live Birth Information Systems have been used. The relative risk of fetal death was calculated, and descriptive statistics and the chi-square test were applied to compare proportions by five-year period. For the spatial analysis, the spatial scanning statistics technique was applied.

**Results:**

A total of 15,336 fetal deaths were recorded, being that 8,132 (53%) were in the first five-year period (2013 to 2017). The variables maternal age, maternal education, type of delivery, fetal sex and birth weight, death in relation to delivery, and place of death, have been related to the five-year periods. Health regions V, VI, VII, and XI presented clusters with a high risk of death.

**Conclusion:**

The characterization of fetal deaths allows us to understand the circumstances that led to these deaths. The clusters with the highest risk of death indicate priority regions for health planning interventions to improve the maternal and child care network.

## INTRODUCTION

Fetal mortality is an indicator of obstetric care, reflecting the comprehensive care provided to women and the quality of health care offered during the prenatal period and during labor^(1)^. The spatial distribution of fetal deaths may reveal inequalities by indicating difficulties in accessing the maternal and child health care network among certain population groups, particularly those with greater social vulnerability^(2)^.

Worldwide, an estimated 2 million fetal deaths occur annually. Of these, 98% are concentrated in low- and middle-income countries, primarily in Africa and Latin America^(3)^. The global fetal mortality rate (FMR) between 2000 and 2021 decreased from 21.3 deaths per 1,000 births to 13.9, representing a 34.8% reduction^(4)^.

In Brazil, 12.1 fetal deaths per 1,000 births were recorded in the year 2000, and 10.8 in 2021, representing a 10.7% decline. The Northeast region, which comprises most of the population with limited access to basic services, reported a rate of 12.1 in 2000 and 12.4 in 2021, showing a 2.5% increase during this period^(5)^.

The more pronounced reduction in the fetal mortality rate (FMR), particularly in socioeconomically vulnerable regions, may have been influenced by the invisibility of fetal deaths, which continue to be treated as secondary issues within maternal and child health policies^(6)^. The importance of the decline in the fetal mortality indicator has been globally recognized beginning in 2014, after it was included in the World Health Organization’s (WHO) *Every Newborn Action Plan* ^(7)^. This plan aims to improve the quality of health care provided to all women and newborns. Among the agreed-upon goals is the reduction of the fetal mortality rate (FMR) to 12 deaths per 1,000 births by the year 2030^(7)^. The plan also recommends expanding the coverage of maternal and child health care services, which may facilitate access to health care for the most vulnerable populations and contribute to reducing regional inequalities in fetal mortality^(8)^.

The unequal distribution of mortality across the territory may be associated with situations of vulnerability arising from geographic, socioeconomic, and cultural factors, which are linked to the living conditions of population groups and influence the education, employment, income, and health status of individuals and communities^(9)^. The development of research employing spatial analysis may contribute to a better understanding of the conditions that influence the occurrence of fetal deaths within the territory.

Spatial analysis makes it possible to integrate geographically referenced socioeconomic, environmental, demographic, and epidemiological characteristics, and it provides visualization, exploratory analysis, and modeling of these data^(10)^. Research employing spatial analysis enables the detection of spatial patterns in fetal mortality and the monitoring of the effectiveness of actions proposed to reduce deaths^(11)^. Through this analysis, it is also possible to identify areas at higher risk for fetal death and, therefore, provide support for the planning of interventions in territories that require priority attention from health managers^(12)^.

Therefore, the objective of this study is to describe the epidemiological characteristics and the use of the spatial scanning technique for the identification of clusters of fetal deaths in Pernambuco between 2013 and 2022.

## METHOD

### Study Design

An ecological study was carried out that used the municipalities of residence as the unit of analysis.

### Location, Population and Selection Criteria

**Figure 1:**
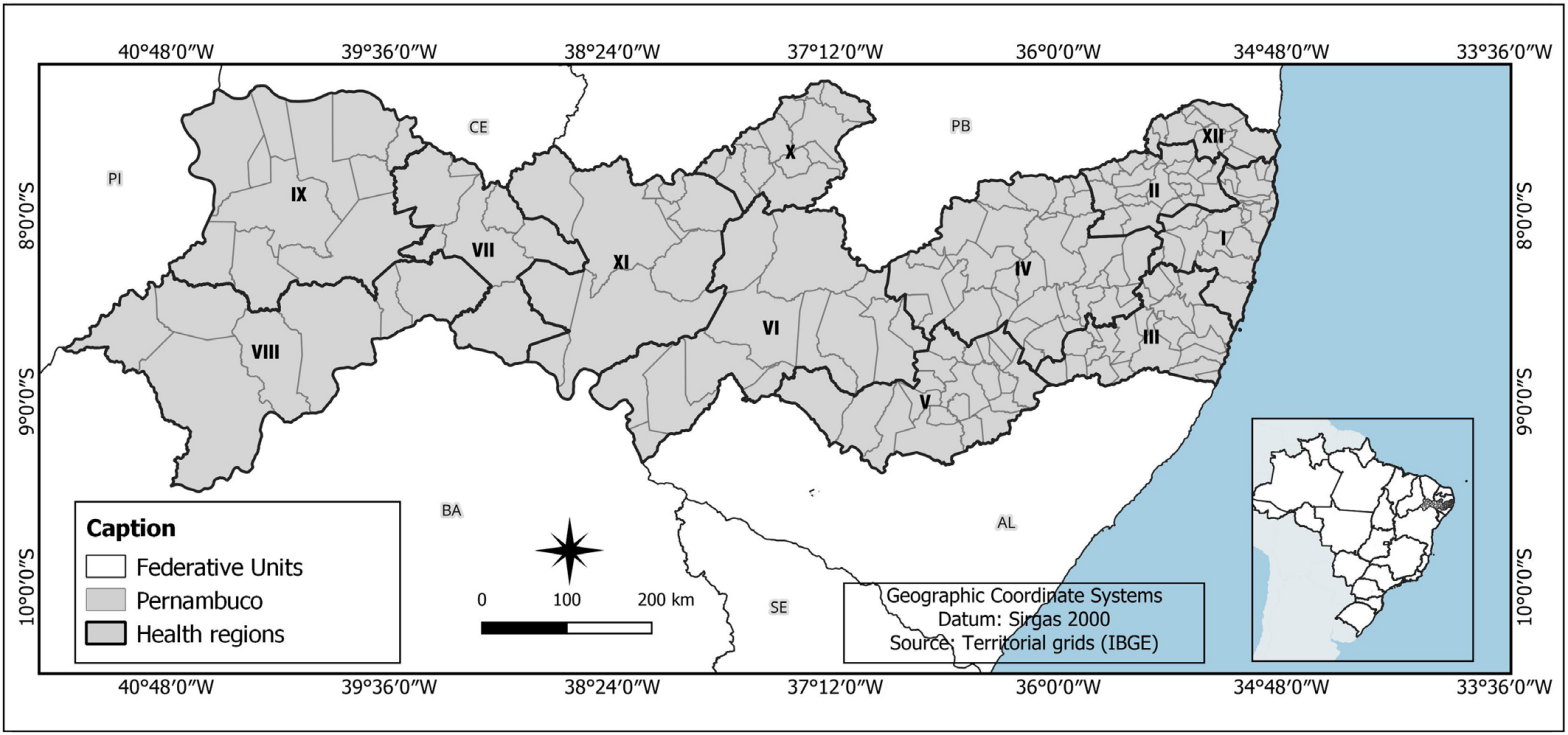
Distribution of municipalities by Health Regions. Pernambuco, 2025.

The study was carried out in the state of Pernambuco, which has a territorial extension of 98,067.877 km^2^ and is home to a population of 9,058,931 inhabitants in 184 municipalities distributed in 12 health regions: I (19 municipalities), II (20 municipalities), III (22 municipalities), IV (32 municipalities), V (21 municipalities), VI (13 municipalities), VII (7 municipalities), VIII (7 municipalities), IX (11 municipalities), X (12 municipalities), XI (10 municipalities) and XII (10 municipalities) (Figure 1)^(13)^.

Prepared by: Author.

To carry out this study, all fetal deaths of mothers residing in Pernambuco, registered in the Mortality Information System from 2013 to 2022, were included.

### Data Collection

Data on fetal deaths registered in the Mortality Information System, having as a reference the study period, were taken from death certificates and used. For the calculation of the relative risk (RR), it was also necessary to include live births to mothers residing in Pernambuco, recorded in the Live Birth Information System during the same period, sourced from live birth certificates^(5)^.

Two study periods have been used: 2013 to 2017 (first five-year period) and 2018 to 2022 (second five-year period). The choice of the analysis period was based on the availability of the database, with 2022 being the most recent year available for consultation at the time of this data analysis. Data were aggregated by five-year period in order to ensure greater stability in the analyses.

The data used were accessed on December 20, 2024 and are available on the electronic platform of the Department of Information Technology of the Unified Health System (DATASUS) (https://datasus.saude.gov.br/informacoes-de-saude-tabnet/).

### Data Analysis and Processing

Variables related to the mother’s characteristics have been analyzed: mother’s age in years (<20, 20-34, and >34), mother’s education in years (<8 and ≥8), type of pregnancy (single, twin, or multiple), weeks of pregnancy (<37 and ≥37), type of delivery (vaginal and cesarean). In addition to variables related to the characteristics of the fetus: gender (male and female), birth weight (<2,500g and ≥2,500g), and place of death (healthcare facility and other locations).

Descriptive statistics and a chi-square test with a significance level <0.05 were applied to compare proportions using the R program, version 3.6, for fetal deaths grouped into five-year periods (2013–2017 and 2018–2022). Using the freely available SatScan 8.2.1 program, which allows the detection of spatial clusters, the relative risk (RR) was calculated and a spatial analysis was performed, using the spatial scanning statistics technique. This technique creates circular windows of variable sizes, whose center moves on the map surface so that it encompasses a set of close neighbors that correspond to a geographic area^(14)^. As the radius moves through all centroids, it varies continuously from zero to the maximum radius, which in this study included up to 10% of the total population. The likelihood function was maximized over all windows, and the most likely cluster was identified. The likelihood ratio for this window constituted the maximum likelihood ratio statistic.

The probabilistic model adopted was the Poisson type, in which, to confirm spatial clusters of fetal deaths, the null hypothesis considered was that the total number of fetal deaths in the state are distributed homogeneously across all municipalities, being proportional to the total number of births (fetal deaths and live births). The alternative hypothesis was that the total number of fetal deaths is distributed heterogeneously, resulting in spatial clusters of deaths.

The distribution under the null hypothesis and the corresponding simulated p-value were obtained by repeating the same analytical exercise, on a large number of random replicates (999) of the data set generated under the null hypothesis, in a Monte Carlo simulation^(14)^. The null hypothesis was rejected when p < 0.05 for the most likely cluster, indicating that in that region it was observed that the risk of fetal death among total births is greater than the expected risk in the same region.

### Ethical Aspects

The study did not require approval from a Research Ethics Committee, in accordance with Resolution No. 674/22 of the National Health Council^(15)^, because secondary data from the public domain, aggregated, were used, and they do not allow individual identification.

## RESULTS

During the studied period, 15,336 fetal deaths were recorded, 8,132 (53%) in the first five-year period (2013 to 2017) and 7,204 (47%) in the second (2018 to 2022). The comparison of the characteristics of delivery and birth of fetal deaths that occurred in the first and second five-year periods showed a statistically significant difference for the variables mother’s age (p < 0.001), mother’s education (p < 0.001), type of delivery (p = 0.001), sex (p = 0.019) and birth weight (p = 0.03) (Table 1).

**Table 1:** Characteristics of fetal deaths by five-year period between 2013 and 2022 – Pernambuco, Brazil.

Variables (n 15.336)	Fetal death		*p-value*
	2013–2017 n (%)	2018–2022 n (%)
**Mother’s age [n = 13.988 (a)]**			**<0.001**
<20	1,557 (57.5)	1,150 (42.5)	
20–34	4,630 (52.8)	4,146 (47.2)	
>34	1,207 (48.2)	1,298 (51.8)	
**Mother’s education [n = 12.578 (b)]**			**<0.001**
<8	3,458 (59.3)	2,371 (40.7)	
≥8	3,279 (48.6)	3,470 (51.4)	
**Type of pregnancy [n = 14.746 (c)]**			**0.305**
Single	7,351 (52.9)	6,547 (47.1)	
Double or more	443 (52.2)	405 (47.8)	
**Weeks of pregnancy [n = 13.445 (d)]**			**0.082**
<37	5,010 (50.9)	4,824 (49.1)	
≥37	1,906 (52.6)	1,715 (47.4)	
**Type of delivery [n = 14.616 (e)]**			**0.001**
Vaginal	5,918 (53.6)	5,126 (46.4)	
Cesarian	1,804 (50.5)	1,768 (49.5)	
**Sex [n = 14.855 (f)]**			**0.020**
Male	4,086 (52.1)	3,761 (47.9)	
Female	3,783 (54)	3,225 (46)	
**Birth weight [n = 14.243 (g)]**			**0.029**
<2.500g	5,333 (52.4)	4,850 (47.6)	
≥2.500g	2,209 (54.4)	1,852 (45.6)	
**Death in relation to delivery [n = 13.309 (h)]**			**<0.001**
Before delivery	7,352 (53.4)	6,422 (46.6)	
During delivery	329 (44.4)	412 (55.6)	
**Place of death [n = 15.258 (i)]**			**<0.001**
Health establishment	7,832 (53.3)	6,856 (46.7)	
Others	262 (46)	308 (54)	

Number/percentage of ignored

(a) 1,348/8.8%

(b) 2,758/18%

(c) 590/3.9%

(d) 1,881/12.3%

(e) 720/4.7%

(f) 481/3.4%

(g) 1,093/7.1%

(h) 825/5.4%

(i) 78/0.5%

The characteristics of mothers’ age being less than 20 years (n = 1,557; 57.5%) and education less than 8 years (n = 3,458; 59.3%) were observed in greater numbers in the first five-year period. Vaginal delivery was observed in greater numbers in the first five-year period (n = 5,918; 53.6%). Regarding the fetus, the male sex (n = 4,086; 52.1%) and birth weight less than 2,500 g (n = 5,332; 52.4%) were more frequent in the first five-year period. Regarding the characteristics of the place of death, a statistically significant difference was observed (<0.001), with a higher occurrence of deaths during childbirth (n = 412; 55.6%) and outside health establishments in the second five-year period (n = 308; 54%) (Table 1).

**Figure 2:**
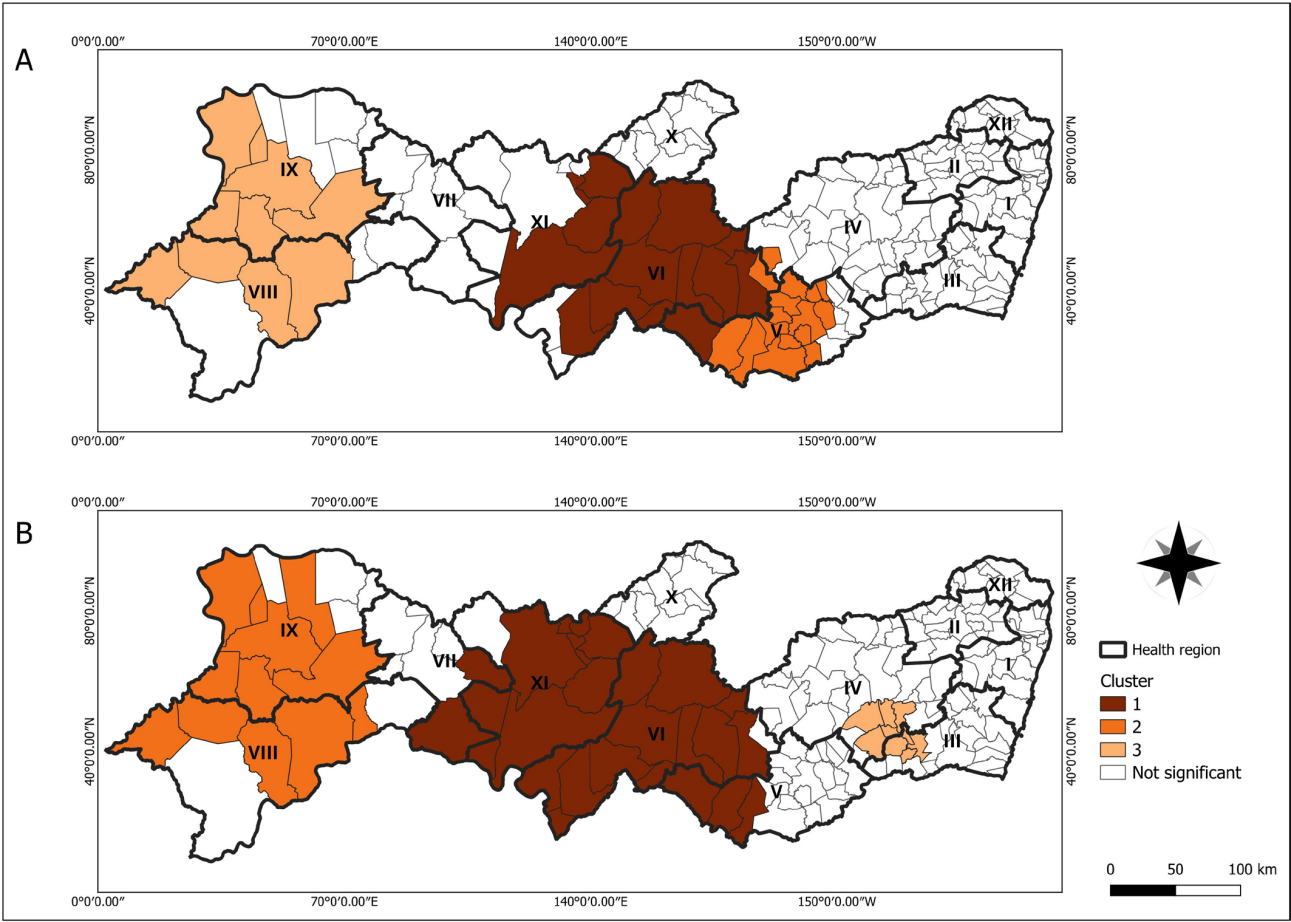
Spatial clusters of risk for fetal death (n = 15,336), Pernambuco, Brazil, 2013–2017 (A); 2018–2022 (B).

Spatial clusters were identified for each of the periods studied. In the first five-year period (2013–2017), three statistically significant clusters have been identified, comprising 41 municipalities in the *Agreste* and *Sertão* regions of Pernambuco, which together accounted for 14% of total births and 17.9% of fetal deaths. Notably, Cluster 1, located predominantly in the *Sertão* region of the state and composed of 15 municipalities belonging to Health Regions V (1 municipality), VI (10), and XI (4), presented a 50% higher risk of fetal death compared to the other municipalities (Figure 2, Table 2).

**Table 2:** Characteristics of spatial clusters of fetal deaths (n = 15,336) – Pernambuco, Brazil, 2013–2017; 2018–2022.

	Number of Deaths							
	Cluster	Number of Municipalities	Observed	Expected	% Deaths	% Population	Relative Risk (RR)	p-value
2013–2017	1	15	549	365	6.8	5.0	1.50	<0.001
	2	16	485	396	6.0	5.0	1.24	0.005
	3	10	409	328	5.1	4.0	1.26	0.006
2018–2022	1	26	829	615	11.6	9.0	1.39	<0.001
	2	12	431	349	6.0	5.0	1.25	0.007
	3	9	190	141	2.6	2.0	1.36	0.04

In the second five-year period (2018–2022), three statistically significant clusters have also been identified, comprising 47 municipalities located in the *Sertão*, *Agreste*, and *Zona da Mata* regions of Pernambuco. These clusters accounted for 15.4% of the state’s total births and concentrated 20.2% of fetal deaths (Figure 2, Table 2). Cluster 1, composed of 26 municipalities located in Health Regions V (3 municipalities), VI (12), VII (2), and XI (9), showed a 39% higher risk of fetal death among total births compared to other municipalities (Figure 2, Table 2).

## DISCUSSION

During the time studied, most fetal deaths occurred in the first five-year period. Maternal characteristics such as age and educational level, as well as fetal characteristics related to type of delivery, sex, birth weight, and place of death, were associated with the analyzed period. Fetal deaths among mothers under 20 years of age, with low educational achievements, and those born through vaginal delivery, male, and with low birth weight occurred mainly in the first five-year period. Deaths that occurred outside health care facilities were more frequent in the second five-year period. Health Regions V, VI, and XI presented clusters with a high risk of fetal death in both five-year periods analyzed.

In this study, a higher percentage of fetal deaths was observed among younger mothers, particularly during the first five-year period analyzed. A study conducted in Vietnam indicates that early pregnancy increases the risk of maternal mortality and contributes to obstetric complications that may lead to fetal death, resulting from restricted intrauterine growth, premature delivery, and low birth weight^(16)^.

Low maternal educational achievement has been observed mainly in the first five-year period. A study conducted in the municipality of Rio de Janeiro found higher mortality rates among children of mothers with low educational levels compared to those of mothers with higher education, particularly concerning fetal death^(17)^.

In this study, the majority of deliveries were vaginal. Similarly, research conducted in Asia demonstrated a higher frequency of fetal deaths among those born through vaginal delivery^(1)^. The literature recommends this type of delivery when maternal and fetal survival conditions are favorable, as it reduces the risk of prematurity, placental complications, and uterine rupture in future pregnancies^(18)^.

However, in emergencies resulting from obstetric complications, the choice of vaginal delivery must be carefully evaluated, as it may jeopardize the survival of both the mother and the baby^(19)^. As observed in a case-control study, cesarean delivery, compared to vaginal delivery, can reduce by half the risk of fetal death^(1)^.

Most deaths occurred among male fetuses. A study conducted in the municipality of São Paulo also found that fetal mortality predominantly affects male infants^(20)^. This finding may be related to the fact that lung development occurs more slowly in male individuals compared to female individuals. Delayed pulmonary maturation increases susceptibility to respiratory infections that can lead to death^(21)^.

Mortality was higher among low birth weight fetuses. The literature indicates that as birth weight decreases, the risk of fetal death increases^(20)^. To reduce the incidence of inadequate birth weight, timely access to prenatal care during the first trimester of pregnancy is essential, with a frequency of six or more visits and provision of quality care^(22)^. During prenatal care, the offering of vaccinations, the performance of laboratory tests, timely diagnosis, and appropriate treatment can contribute to the reduction of fetal mortality^(23)^.

Regarding the place of death, in the present study, fetal deaths occurring outside health care facilities were observed mainly in the second five-year period analyzed. According to a study conducted in Bangladesh, timely access to delivery care services can reduce early fetal and neonatal mortality by half^(24)^. This is because delays and travel during labor contribute to deaths, resulting from inequalities in access to obstetric health services, which predominantly affect pregnant women with greater socioeconomic vulnerability, according to a national study^(2)^.

When examining fetal deaths that occurred outside health care facilities in the second five-year period of this study (2018–2022), it is important to consider the COVID-19 pandemic, which was declared in 2020^(25)^. A study conducted in India indicates that delays in pregnant women reaching health care services, late performance of operative procedures, and the need to travel between facilities for delivery increased the risk of fetal death during the pandemic period^(26)^. It is well known that during the pandemic, in order to prioritize care for COVID-19 cases, in-person prenatal services were interrupted and obstetric care was reduced, which may have contributed to the occurrence of fetal deaths^(26)^.

The results of the spatial scanning analysis allowed the identification, in both five-year periods analyzed, of clusters with a higher risk of fetal death in Health Regions V, VI, and XI. In the second five-year period, the emergence of a cluster in Region VII was also observed. In addition to exhibiting high fetal mortality, municipalities in Regions V, VI, and XI are also considered priority areas for the implementation of actions aimed at reducing social deprivation, according to a previous study^(27)^.

The presence of fetal mortality clusters in certain regions may also indicate weaknesses in the Pernambuco Maternal and Child Health Network (Rede Materno Infantil/Pernambuco/Rede Cegonha), which aims to ensure women’s rights to reproductive planning and to humanized care during pregnancy, delivery, and the puerperium, as well as children’s rights to a safe birth. Among the gaps observed in the network, it is noted that in municipalities of Health Region V, there is no identifiable reference maternity hospital for high-risk pregnancies. In Regions VI and XI, there are no high-risk pregnancy beds or neonatal intensive care unit (NICU) beds. The reference maternity hospital for high-risk pregnancies located in Region VII, although it has high-risk pregnancy beds, does not have a neonatal ICU^(28)^.

Similarly to the present study, spatial analysis has been used in the literature to indicate the distribution of fetal deaths across territories, as observed in a study conducted in Uganda^(29)^. The analysis of georeferenced data can also support the understanding of local contexts and factors associated with the occurrence of deaths, as observed in a study conducted in Mozambique^(30)^. Furthermore, it allows the identification of areas that should be prioritized in decision-making processes aimed at reducing the fetal mortality rate. Through spatial analysis, it is possible to monitor the effectiveness of public policies, as demonstrated in a study that analyzed the quality of primary care services and the reduction of infant mortality in Brazil^(12)^.

Therefore, the importance of conducting new research that assesses the risk of fetal death in different territories, identifies factors associated with the spatial distribution of mortality, highlights areas that should be prioritized for health interventions, and monitors actions aimed at improving health indicators is reinforced. The limitations of this study may be related to the use of secondary data, possible underreporting of fetal deaths, and incompleteness of health information systems, which may result in an underestimation of the calculated risks. Additionally, the unit of analysis may obscure intracity inequalities.

The results of this study may contribute to nursing by identifying areas with a higher risk of fetal death. By addressing this public health issue and providing support for decision-making, this research may guide the allocation of resources to strengthen care practices that enable the reduction of preventable deaths.

## CONCLUSION

The characterization of fetal deaths presented in the results of this study contributes to the understanding of the circumstances that led to their occurrence. The variables that were statistically significant in the five-year periods analyzed included: maternal age, maternal educational level, type of delivery, sex, birth weight, death in relation to delivery, and place of death. Spatial analysis identified clusters with a higher risk of fetal death in Health Regions V, VI, VII, and XI. This analysis also allowed the identification of areas that require intervention through health planning, with immediate actions aimed at improving the maternal and child health care network.

## Data Availability

The entire dataset supporting the results of this study was published in the article itself.
