# Supplementary material for: Spatial scanning for the identification of risk areas for fetal death: an ecological study in Pernambuco
Source: Rev Esc Enferm USP. 2026 Jan 9;59:e20250248. doi: 10.1590/1980-220X-REEUSP-2025-0248en (PMC12810357; doi:10.1590/1980-220X-REEUSP-2025-0248en)
Supplement: Translation

# Translation: Varredura espacial para identificação de áreas de risco do óbito fetal: estudo ecológico em Pernambuco

**DOI:** 10.1590/1980-220X-REEUSP-2025-0248pt

**Authors:** Indianara Maria de Barros Canuto, Cristine Vieira do Bonfim, Amanda Priscila de Santana Cabral Silva

**Affiliations:** 1 Fundação Oswaldo Cruz, Instituto Aggeu Magalhães, Recife, PE, Brasil.; 2 Secretaria de Saúde do Recife, Distrito Sanitário II, Recife, PE, Brasil.; 3 Fundação Joaquim Nabuco, Recife, PE, Brasil.; 4 Universidade Federal de Pernambuco, Curso de Bacharelado em Saúde Coletiva, Vitória de Santo Antão, PE, Brasil.

**Keywords:** Mortalidade Fetal, Estatísticas Vitais, Sistemas de Informação em Saúde, Análise Espacial, Monitoramento Epidemiológico.

## Abstract

**Objetivo:**

Descrever as características epidemiológicas e a utilização da técnica de varredura espacial para a identificação de *clusters* de óbitos fetais em Pernambuco, entre 2013 e 2022.

**Método:**

Estudo ecológico que teve os municípios como unidade de análise, realizado em Pernambuco. Utilizaram-se dados dos Sistemas de Informações sobre Mortalidade e sobre Nascidos Vivos. Calculou-se o risco relativo ao óbito fetal e aplicou-se estatística descritiva e teste qui-quadrado para comparação de proporções por quinquênio. Para a análise espacial, aplicou-se a técnica de estatística de varredura espacial.

**Resultados:**

Registraram-se 15.336 óbitos fetais, sendo 8.132 (53%) no primeiro quinquênio (2013 a 2017). As variáveis idade da mãe, escolaridade da mãe, tipo de parto, sexo e peso do feto ao nascer, óbito em relação ao parto e local de ocorrência do óbito estiveram relacionadas aos quinquênios. As regiões de saúde V, VI, VII e XI apresentaram *clusters* com elevado risco ao óbito.

**Conclusão:**

A caraterização dos óbitos fetais permite compreender circunstâncias que implicaram na ocorrência das mortes. Os *clusters* de maior risco ao óbito apontam regiões prioritárias à intervenção do planejamento em saúde para melhoria da rede de assistência materna e infantil.

## INTRODUÇÃO

A mortalidade fetal é um indicador da assistência obstétrica, que reflete o cuidado integral à mulher e a qualidade da atenção à saúde ofertada durante o período pré-natal e no momento do parto^(1)^. A distribuição espacial dos óbitos fetais pode demonstrar desigualdades ao apontar dificuldades no acesso à rede de saúde materna e infantil por determinados grupos populacionais, sobretudo aqueles com maior vulnerabilidade social^(2)^.

No mundo, estima-se a ocorrência de 2 milhões de mortes fetais anuais. Destas, 98% localizam-se em países de baixa e média renda, principalmente na África e América Latina^(3)^. A taxa de mortalidade fetal (TMF) mundial no período de 2000 a 2021 passou de 21,3 óbitos por 1.000 nascimentos para 13,9, com redução de 34,8%^(4)^.

No Brasil, foram registrados 12,1 óbitos fetais a cada 1.000 nascimentos no ano 2000, e 10,8 em 2021, com declínio de 10,7%. A região Nordeste, que apresenta a maior parte da população com acesso deficitário a serviços básicos, apresentou taxa de 12,1 no ano 2000 e de 12,4 em 2021, com aumento de 2,5% nesse período^(5)^.

A redução mais acentuada da TMF, principalmente nas regiões socioeconomicamente vulneráveis, pode ter sofrido influência da invisibilidade dos óbitos fetais, que permanecem secundarizados nas políticas de saúde materna e infantil^(6)^. A importância do declínio do indicador de mortalidade fetal foi apresentada globalmente a partir do ano de 2014, após ser inserida no *The Every Newborn Action Plan* da Organização Mundial da Saúde (OMS)^(7)^. Com esse plano, pretende-se aperfeiçoar a qualidade dos cuidados em saúde fornecidos para todas as mulheres e recém-nascidos. Entre as metas pactuadas, está reduzir, até 2030, a TMF para 12 óbitos por mil nascimentos^(7)^. O plano também recomenda que a cobertura da assistência à saúde materna e infantil seja aumentada. O que poderá favorecer o acesso aos cuidados de saúde pelas populações mais vulneráveis e contribuir com a redução das desigualdades regionais da mortalidade fetal^(8)^.

A distribuição desigual da mortalidade no território pode estar relacionada à situação de vulnerabilidade decorrente de aspectos geográficos, socioeconômicos e culturais, que estão relacionados à vida nos grupos populacionais e influenciam as condições de educação, emprego, renda e saúde dos indivíduos e da coletividade^(9)^. A elaboração de pesquisas que façam uso da análise espacial poderá contribuir para uma melhor compreensão das condições que interferem na ocorrência dos óbitos fetais no território.

A análise espacial possibilita agregar características socioe-conômicas, ambientais, demográficas e epidemiológicas referenciadas geograficamente, e proporciona a visualização, a análise exploratória e a modelagem desses dados^(10)^. Pesquisas com análise espacial possibilitam a detecção de padrões espaciais da mortalidade fetal e o monitoramento da efetividade das ações propostas para a redução dos óbitos^(11)^. Por meio dessa análise, também é possível identificar áreas com maior risco à ocorrência do óbito fetal e, assim, fornecer subsídios para o planejamento de ações em territórios que necessitam de atenção prioritária dos gestores da saúde^(12)^.

Dessa forma, o objetivo deste estudo é descrever as características epidemiológicas e a utilização da técnica de varredura espacial para a identificação de *clusters* de óbitos fetais em Pernambuco, entre 2013 e 2022.

## MÉTODO

### Desenho do Estudo

Foi realizado um estudo ecológico que utilizou os municípios de residência como unidade de análise.

### Local, População e Critérios de Seleção

O estudo foi realizado no estado de Pernambuco, que apresenta uma extensão territorial de 98.067,877 km^2^ e abriga uma população de 9.058.931 habitantes em 184 municípios distribuídos em 12 regiões de saúde: I (19 municípios), II (20 municípios), III (22 municípios), IV (32 municípios), V (21 municípios), VI (13 municípios), VII (7 municípios), VIII (7 municípios), IX (11 municípios), X (12 municípios), XI (10 municípios) e XII (10 municípios) (Figura 1)^(13)^.

**Figura 1:** Distribuição dos municípios por Regiões de Saúde. Pernambuco, 2025.

Elaboração: Autor.

Para a realização deste estudo, foram incluídos todos os óbitos fetais de mães residentes em Pernambuco, registrados no Sistema de Informações sobre Mortalidade (SIM) no período de 2013 a 2022.

### Coleta de Dados

Foram utilizados dados dos óbitos fetais registrados no SIM, alimentados pela declaração de óbito, referentes ao período estudado. Para o cálculo do risco relativo (RR), também foi necessária a inclusão dos nascidos vivos de mães residentes em Pernambuco e registrados no Sistema de Informações sobre Nascidos Vivos (Sinasc) no mesmo período analisado, alimentado pela declaração de nascidos vivos^(5)^.

Foram utilizados dois períodos de estudo: 2013 a 2017 (primeiro quinquênio) e 2018 a 2022 (segundo quinquênio). A escolha do período de análise baseou-se na disponibilidade do banco de dados, cujo ano de 2022 era o mais recente disponível para consulta no momento da análise dos dados. Foi realizada agregação dos dados por quinquênio para garantir maior estabilidade nas análises.

Os dados utilizados foram acessados em 20 de dezembro de 2024 e encontram-se disponíveis na plataforma eletrônica do Departamento de Informática do Sistema Único de Saúde (DATASUS) (https://datasus.saude.gov.br/informacoes-de-saude-tabnet/).

### Análise e Tratamento dos Dados

Foram analisadas variáveis referentes às características da mãe: idade da mãe em anos (<20, 20-34 e >34), escolaridade da mãe em anos (<8 e ≥8), tipo de gravidez (única e dupla ou mais), semanas de gestação (<37 e ≥37), tipo de parto (vaginal e cesáreo). Além das variáveis referentes às características do feto: sexo (masculino e feminino), peso ao nascer (<2.500g e ≥2.500g) e local de ocorrência do óbito (estabelecimento de saúde e outros locais).

Aplicou-se estatística descritiva e teste qui-quadrado com nível de significância <0,05 para comparação de proporções por meio do programa R, versão 3.6., para os óbitos fetais agrupados em quinquênios (2013–2017 e 2018–2022). Por meio do programa de acesso gratuito SatScan 8.2.1, que permite detectar aglomerados espaciais, calculou-se o risco relativo (RR) e foi realizada análise espacial, utilizando-se da técnica de estatística de varredura espacial. Essa técnica cria janelas circulares de tamanhos variáveis, cujo centro se move na superfície do mapa de modo que englobe um conjunto de vizinhos próximos que correspondem a uma área geográfica^(14)^. Ao se mover por todos os centroides, o raio varia continuamente de zero ao raio máximo, que neste estudo incluiu até 10% da população total. A função de probabilidade foi maximizada sobre todas as janelas e identificou-se o agrupamento mais provável. A razão de probabilidade para essa janela constituiu o teste estatístico da razão de probabilidade máxima.

O modelo probabilístico adotado foi do tipo Poisson, em que, para a confirmação de aglomerados espaciais de óbitos fetais, considerou-se como hipótese nula que o total de óbitos fetais do estado são distribuídos de forma homogênea em todos os municípios, sendo proporcional aos nascimentos totais (óbitos fetais e nascidos vivos). A hipótese alternativa foi a de que o número total de óbitos fetais se distribui de maneira heterogênea, resultando em aglomerados espaciais de óbitos.

A distribuição sob a hipótese nula e o valor de p simulado correspondente foi obtida pela repetição do mesmo exercício analítico, num grande número de réplicas aleatórias (999) do conjunto de dados gerados sob a hipótese nula, numa simulação do tipo Monte Carlo^(14)^. A hipótese nula foi rejeitada quando p < 0,05 para o aglomerado mais provável, indicando que naquela região foi observado que o risco de óbito fetal entre os nascidos totais é maior do que o risco esperado na mesma região.

### Aspectos Éticos

O estudo não necessitou de aprovação de um Comitê de Ética em Pesquisa, conforme a Resolução nº 674/22 do Conselho Nacional de Saúde^(15)^, pois foram utilizados dados secundários de domínio público, agregados, que não possibilitam a identificação individual.

## RESULTADOS

No período estudado, foram registrados 15.336 óbitos fetais, sendo 8.132 (53%) no primeiro quinquênio (2013 a 2017) e 7.204 (47%) no segundo (2018 a 2022). A comparação das características do parto e nascimento dos óbitos fetais ocorridos no primeiro e segundo quinquênio mostrou diferença estatisticamente significante para as variáveis idade da mãe (p < 0,001), escolaridade da mãe (p < 0,001), tipo de parto (p = 0,001), sexo (p = 0,019) e peso ao nascer (p = 0,03) (Tabela 1).

**Tabela 1:** Características dos óbitos fetais por quinquênio entre 2013 e 2022 – Pernambuco, Brasil.

Variáveis (n 15.336)	Óbito fetal		*p-valor*
	2013–2017 n (%)	2018–2022 n (%)	
**Idade da mãe [n = 13.988 (a)]**			**<0,001**
<20	1.557 (57,5)	1.150 (42,5)	
20–34	4.630 (52,8)	4.146 (47,2)	
>34	1.207 (48,2)	1.298 (51,8)	
**Escolaridade da mãe [n = 12.578 (b)]**			**<0,001**
<8	3.458 (59,3)	2.371 (40,7)	
≥8	3.279 (48,6)	3.470 (51,4)	
**Tipo de gravidez [n = 14.746 (c)]**			**0,305**
Única	7.351 (52,9)	6.547 (47,1)	
Dupla ou mais	443 (52,2)	405 (47,8)	
**Semanas de gestação [n = 13.445 (d)]**			**0,082**
<37	5.010 (50,9)	4.824 (49,1)	
≥37	1.906 (52,6)	1.715 (47,4)	
**Tipo de parto [n = 14.616 (e)]**			**0,001**
Vaginal	5.918 (53,6)	5.126 (46,4)	
Cesário	1.804 (50,5)	1.768 (49,5)	
**Sexo [n = 14.855 (f)]**			**0,020**
Masculino	4.086 (52,1)	3.761 (47,9)	
Feminino	3.783 (54)	3.225 (46)	
**Peso ao nascer [n = 14.243 (g)]**			**0,029**
<2.500g	5.333 (52,4)	4.850 (47,6)	
≥2.500g	2.209 (54,4)	1.852 (45,6)	
**Óbito em relação ao parto [n = 13.309 (h)]**			**<0,001**
Antes do parto	7.352 (53,4)	6.422 (46,6)	
Durante o parto	329 (44,4)	412 (55,6)	
**Local de ocorrência do óbito [n = 15.258 (i)]**			**<0,001**
Estabelecimento de saúde	7.832 (53,3)	6.856 (46,7)	
Outros	262 (46)	308 (54)	

Número/percentual de ignorados

(a) 1.348/8,8%

(b) 2.758/18%

(c) 590/3,9%

(d) 1.881/12,3%

(e) 720/4,7%

(f) 481/3,4%

(g) 1.093/7,1%

(h) 825/5,4%

(i) 78/0,5%

As caraterísticas maternas de idade inferior a 20 anos (n = 1.557; 57,5%) e escolaridade menor que 8 anos (n = 3.458; 59,3%) foram verificadas em maior número no primeiro quinquênio. O parto vaginal foi observado em quantitativo superior no primeiro quinquênio (n = 5.918; 53,6%). Em relação ao feto, o sexo masculino (n = 4.086; 52,1%) e o peso ao nascer inferior a 2.500 g (n = 5.332; 52,4%) apresentaram maior quantitativo no primeiro quinquênio. Com relação às características do local do óbito, verificou-se diferença estatisticamente significante (<0,001), com maior ocorrência de óbitos durante o parto (n = 412; 55,6%) e fora dos estabelecimentos de saúde no segundo quinquênio (n = 308; 54%) (Tabela 1).

Foram identificados aglomerados espaciais em cada um dos períodos estudados. No primeiro quinquênio (2013–2017), foram identificados três *clusters* estatisticamente significantes, formados por 41 municípios do Agreste e Sertão de Pernambuco, que juntos concentraram 14% dos nascimentos totais e abarcaram 17,9% dos óbitos fetais. Destaca-se o *cluster* 1, localizado predominantemente no Sertão do estado, formado por 15 municípios pertencentes às regiões de saúde V (1 município), VI (10) e XI (4), que tiveram um risco 50% maior de óbito fetal que nos demais municípios (Figura 2, Tabela 2).

No segundo quinquênio (2018–2022), também foram identificados três *clusters* estatisticamente significantes, formados por 47 municípios localizados no Sertão, Agreste e na Zona da Mata pernambucana. Esses agregados respondem por 15,4% dos nascimentos totais do estado e concentraram 20,2% dos óbitos fetais (Figura 2, Tabela 2). O *cluster* 1, formado por 26 municípios localizados nas regiões de saúde V (3 municípios), VI (12), VII (2), XI (9). Nesse *cluster*, o risco dos nascimentos totais configurarem óbito fetal foi 39% maior se comparado a outros municípios (Figura 2, Tabela 2).

**Figura 2:** Aglomerados espaciais de risco ao óbito fetal (n = 15.336), Pernambuco, Brasil, 2013 a 2017 (A); 2018 a 2022 (B).

**Tabela 2:** Características dos aglomerados espaciais de óbitos fetais (n = 15.336) – Pernambuco, Brasil, 2013 a 2017; 2018 a 2022.

	Nº de óbitos							
	*Cluster*	Nº de municípios	Observados	Esperados	% Óbitos	% População	Risco Relativo (RR)	p-valor
2013–2017	1	15	549	365	6,8	5,0	1,50	<0,001
	2	16	485	396	6,0	5,0	1,24	0,005
	3	10	409	328	5,1	4,0	1,26	0,006
2018–2022	1	26	829	615	11,6	9,0	1,39	<0,001
	2	12	431	349	6,0	5,0	1,25	0,007
	3	9	190	141	2,6	2,0	1,36	0,04

## DISCUSSÃO

No período estudado, a maior parte dos óbitos fetais ocorreu no primeiro quinquênio. As características maternas de idade e escolaridade, além das características fetais referentes ao tipo de parto, sexo, peso ao nascer e local de ocorrência do óbito, relacionaram-se ao quinquênio analisado. Os óbitos fetais de mães com idade inferior a 20 anos, baixa escolaridade, e os nascidos por parto vaginal, do sexo masculino e com baixo peso, ocorreram principalmente no primeiro quinquênio. Os óbitos ocorridos fora de estabelecimentos de saúde apresentaram maior quantitativo no segundo quinquênio. As regiões de saúde V, VI e XI apresentaram *clusters* com elevado risco ao óbito fetal nos dois quinquênios analisados.

Neste estudo, observou-se maior percentual de mortes fetais em mães com menos idade, principalmente no primeiro quinquênio analisado. Estudo realizado no Vietnã aponta que a gravidez precoce eleva o risco de mortalidade materna, além de contribuir para a existência de complicações obstétricas que podem levar à morte do feto, decorrentes do crescimento intrauterino reduzido, parto prematuro e baixo peso ao nascer^(16)^.

A baixa escolaridade materna foi visualizada sobretudo no primeiro quinquênio. Pesquisa elaborada no município do Rio de Janeiro, observou maiores taxas de mortalidade de filhos de mãe com baixa escolaridade quando comparados aos filhos de mãe com alta escolaridade, sobretudo para o óbito fetal^(17)^.

Neste estudo, foi observada a maioria dos partos do tipo vaginal. De modo semelhante, pesquisa realizada na Ásia demonstrou maior frequência de óbitos fetais nascidos por parto vaginal^(1)^. A literatura recomenda essa via de parto quando há boas condições de sobrevivência materna e fetal, pois reduz o risco de prematuridade, de complicações placentárias e ruptura uterina em futuras gestações^(18)^.

Entretanto, em emergências devido a complicações obstétricas, a escolha pelo parto vaginal deve ser avaliada, pois pode ameaçar a sobrevivência da mãe e do bebê^(19)^. Conforme observado em estudo de caso-controle, a cesariana, se comparada ao parto vaginal, é capaz de diminuir pela metade as chances de óbito fetal^(1)^.

A maior parte das mortes ocorreu entre os fetos do sexo masculino. Pesquisa realizada no município de São Paulo, também constatou que a mortalidade fetal afeta principalmente o sexo masculino^(20)^. Tal resultado pode estar relacionado ao fato de o desenvolvimento pulmonar ocorrer de maneira mais lenta nos indivíduos do sexo masculino em comparação aos do sexo feminino. O amadurecimento pulmonar tardio acarreta maior suscetibilidade a infecções respiratórias que ocasionam o óbito^(21)^.

A mortalidade foi mais elevada entre fetos com baixo peso ao nascer. A literatura aponta que à medida que o peso ao nascer diminui, o risco de mortalidade fetal se eleva^(20)^. Para reduzir os nascimentos com peso inadequado, é fundamental o acesso oportuno ao pré-natal durante o primeiro trimestre de gestação, com frequência de seis ou mais consultas e assistência de qualidade^(22)^. Durante os cuidados pré-natais, a oferta de vacinação, a realização de exames laboratoriais, o diagnóstico oportuno e o tratamento adequado podem contribuir com o declínio da mortalidade fetal^(23)^.

Quanto ao local de ocorrência do óbito, no presente estudo foram observadas mortes fetais que aconteceram fora dos estabelecimentos de saúde principalmente no segundo quinquênio analisado. De acordo com pesquisa realizada em Bangladesh, o acesso oportuno a serviços de assistência ao parto é capaz de reduzir pela metade a mortalidade fetal e neonatal precoce^(24)^. Pois a peregrinação no momento do parto contribui para a ocorrência de mortes, ocasionadas pela desigualdade no acesso aos serviços de saúde obstétricos, que afetam sobretudo as gestantes com maior vulnerabilidade socioeconômica, de acordo com pesquisa nacional^(2)^.

Ao averiguar as mortes fetais ocorridas fora dos estabelecimentos de saúde no segundo quinquênio deste estudo (2018–2022), é importante considerar a pandemia de covid-19, decretada no ano de 2020^(25)^. Estudo realizado na Índia aponta que a demora da chegada da gestante aos serviços de saúde, a realização tardia de procedimentos operatórios e a peregrinação para a realização do parto aumentaram o risco ao óbito fetal no período pandêmico^(26)^. É sabido que durante a pandemia, para priorizar a assistência aos casos de covid-19, houve interrupção dos serviços presenciais de atenção ao pré-natal e redução dos cuidados obstétricos, o que pode ter contribuído para a ocorrência de mortes fetais^(26)^.

Os resultados da análise de varredura espacial permitiram identificar nos dois quinquênios analisados, *clusters* de maior risco para ocorrência de óbitos fetais nas regiões de saúde V, VI e XI. Para o segundo quinquênio, verificou-se o surgimento de *cluster* na região VII. Além de apresentarem alta mortalidade fetal, municípios das regiões V, VI e XI também são considerados áreas prioritárias para o desenvolvimento de ações voltadas à redução da carência social, de acordo com trabalho anterior realizado^(27)^.

A presença de *clusters* de mortalidade fetal em algumas regiões também pode indicar fragilidades na Rede Materno Infantil de Pernambuco/Rede Cegonha, que visa assegurar à mulher o direito ao planejamento reprodutivo e à atenção humanizada à gravidez, ao parto e ao puerpério, bem como à criança o direito ao nascimento seguro. Entre as lacunas observadas na rede, verifica-se que nos municípios da V região de saúde (RS) não é possível identificar maternidade de referência para gestação de alto risco. Nas regiões VI e XI, não existem leitos de gestação de alto risco e leitos de unidade de terapia intensiva (UTI) neonatal. A maternidade de referência para alto risco localizada na RS VII, apesar de ter leitos de gestação de alto risco, não possui UTI neonatal^(28)^.

De modo semelhante ao presente estudo, a análise espacial tem sido utilizada na literatura para apontar a distribuição das mortes fetais nos territórios, conforme visualizado em pesquisa realizada na Uganda^(29)^. A análise de dados georreferenciados também é capaz de subsidiar a compreensão dos contextos locais e dos fatores associados à ocorrência dos óbitos, como pode ser observado em estudo realizado em Moçambique^(30)^. Além disso, permite identificar áreas que devem ser prioritárias na tomada de decisões que contribuam para a redução da taxa de mortalidade fetal. Por meio da análise espacial, é possível monitorar a eficácia de políticas públicas, o que pode ser identificado em uma pesquisa que analisou a qualidade dos serviços de atenção primária e a redução da mortalidade infantil no Brasil^(12)^.

Assim, reforça-se a importância da elaboração de novas pesquisas que verifiquem o risco à ocorrência de óbitos em diferentes territórios, identifiquem fatores associados à distribuição espacial da mortalidade, apontem áreas prioritárias às intervenções em saúde e monitorem as ações realizadas na melhoria dos indicadores de saúde. As limitações deste estudo podem estar relacionadas à utilização de dados secundários, à possível subnotificação dos óbitos fetais e à incompletude dos sistemas de informações em saúde. O que é capaz de resultar em um subdimensionamento dos riscos calculados. Além disso, a unidade de análise pode ocultar desigualdades intramunicipais.

Os resultados deste estudo podem contribuir para a enfermagem na identificação de áreas com maior risco ao óbito fetal. Pois, ao discutir esse problema de saúde pública e fornecer subsídios à tomada de decisão, esta pesquisa poderá nortear a alocação de recursos para o fortalecimento de práticas assistenciais que possibilitem a redução das mortes evitáveis.

## CONCLUSÃO

A caracterização das mortes fetais apresentadas nos resultados deste estudo contribui para o entendimento das circunstâncias que implicaram na ocorrência dos óbitos. As variáveis estatisticamente significantes nos quinquênios analisados foram: idade da mãe, escolaridade da mãe, tipo de parto, sexo, peso ao nascer, óbito em relação ao parto e local de ocorrência do óbito. A análise espacial verificou *clusters* de risco mais elevado à ocorrência de óbito fetal nas regiões V, VI, VII e XI. Essa análise também permitiu indicar áreas que necessitam de intervenção do planejamento em saúde com ações imediatas destinadas à melhoria da rede de assistência materna e infantil.

## Data Availability

Todo o conjunto de dados que dá suporte aos resultados deste estudo foi publicado no próprio artigo.

# Translation: Escaneo espacial para identificar áreas de riesgo de muerte fetal: estudio ecológico en Pernambuco

**DOI:** 10.1590/1980-220X-REEUSP-2025-0248es

**Authors:** Indianara Maria de Barros Canuto, Cristine Vieira do Bonfim, Amanda Priscila de Santana Cabral Silva

**Affiliations:** 1 Fundação Oswaldo Cruz, Instituto Aggeu Magalhães, Recife, PE, Brasil.; 2 Secretaria de Saúde do Recife, Distrito Sanitário II, Recife, PE, Brasil.; 3 Fundação Joaquim Nabuco, Recife, PE, Brasil.; 4 Universidade Federal de Pernambuco, Curso de Bacharelado em Saúde Coletiva, Vitória de Santo Antão, PE, Brasil.

**Keywords:** Mortalidad fetal, Estadísticas Vitales, Sistemas de Información en Salud, Análisis Espacial, Vigilancia epidemiológica

## Abstract

**Objetivo:**

Describir las características epidemiológicas y el uso de la técnica de escaneo espacial para identificar *clusters* de muertes fetales en Pernambuco, entre 2013 y 2022.

**Método:**

Estudio ecológico con municipios como unidad de análisis, realizado en Pernambuco. Se utilizaron datos de los Sistemas de Información de Mortalidad y Nacidos Vivos. Se calculó el riesgo relativo de muerte fetal y se aplicaron estadísticas descriptivas y la prueba de chi-cuadrado para comparar proporciones por quinquenio. Para el análisis espacial, se aplicó la técnica de estadística de barrido espacial.

**Resultados:**

Se registraron 15.336 muertes fetales, de las cuales 8.132 (53%) ocurrieron en el primer quinquenio (2013–2017). Las variables edad materna, educación materna, tipo de parto, sexo y peso fetal al nacer, muerte relacionada con el parto y lugar de fallecimiento se relacionaron con los quinquenios. Las regiones sanitarias V, VI, VII y XI presentaron conglomerados con alto riesgo de muerte.

**Conclusión:**

La caracterización de las muertes fetales permite comprender las circunstancias que las llevaron a ocurrir. Los conglomerados con mayor riesgo de muerte indican regiones prioritarias para intervenciones de planificación sanitaria destinadas a mejorar la red de atención materno infantil.

## INTRODUCCIÓN

La mortalidad fetal es un indicador de la asistencia obstétrica, que refleja la atención integral a la mujer y la calidad de la atención de salud ofrecida durante el período prenatal y en el momento del parto^(1)^. La distribución espacial de las muertes fetales puede poner de manifiesto las desigualdades al señalar las dificultades de acceso a la red de salud materno-infantil de determinados grupos de población, especialmente los más vulnerables socialmente^(2)^.

Se estima que en el mundo se producen 2 millones de muertes fetales al año. De estas, el 98% se producen en países de ingresos bajos y medios, principalmente en África y América Latina^(3)^. La tasa de mortalidad fetal (TMF) mundial en el periodo comprendido entre 2000 y 2021 pasó de 21,3 muertes por cada 1000 nacimientos a 13,9, lo que supone una reducción del 34,8%^(4)^.

En Brasil, se registraron 12,1 muertes fetales por cada 1000 nacimientos en el año 2000 y 10,8 en 2021, lo que supone un descenso del 10,7%. La región noreste, donde se concentra la mayor parte de la población con acceso deficitario a los servicios básicos, presentó una tasa de 12,1 en el año 2000 y de 12,4 en 2021, con un aumento del 2,5% en ese periodo^(5)^.

La reducción más acusada de la TMF, principalmente en las regiones socioeconómicamente vulnerables, puede haber estado influenciada por la invisibilidad de las muertes fetales, que siguen siendo secundarias en las políticas de salud materna e infantil^(6)^. La importancia de la disminución del indicador de mortalidad fetal se presentó a nivel mundial a partir del año 2014, tras su inclusión en el Plan de Acción para Todos los Recién Nacidos de la Organización Mundial de la Salud (OMS)^(7)^. Con este plan se pretende mejorar la calidad de la atención sanitaria prestada a todas las mujeres y recién nacidos. Entre los objetivos acordados se encuentra la reducción, para 2030, de la TMF a 12 muertes por cada mil nacimientos^(7)^. El plan también recomienda que se aumente la cobertura de la asistencia sanitaria materno-infantil. Esto podría favorecer el acceso a la atención sanitaria de las poblaciones más vulnerables y contribuir a la reducción de las desigualdades regionales en materia de mortalidad fetal^(8)^.

La distribución desigual de la mortalidad en el territorio puede estar relacionada con la situación de vulnerabilidad derivada de aspectos geográficos, socioeconómicos y culturales, que están relacionados con la vida de los grupos poblacionales e influyen en las condiciones de educación, empleo, ingresos y salud de los individuos y la colectividad^(9)^. La elaboración de investigaciones que utilicen el análisis espacial puede contribuir a una mejor comprensión de las condiciones que interfieren en la ocurrencia de muertes fetales en el territorio.

El análisis espacial permite agregar características socioeconómicas, ambientales, demográficas y epidemiológicas referenciadas geográficamente, y proporciona la visualización, el análisis exploratorio y la modelización de estos datos^(10)^. Las investigaciones con análisis espacial permiten detectar patrones espaciales de mortalidad fetal y monitorear la eficacia de las acciones propuestas para reducir las muertes^(11)^. A través de este análisis, también es posible identificar áreas con mayor riesgo de ocurrencia de muerte fetal y, así, proporcionar subsidios para la planificación de acciones en territorios que necesitan atención prioritaria de los gestores de salud^(12)^.

De este modo, el objetivo de este estudio es describir las características epidemiológicas y el uso de la técnica de escaneo espacial para la identificación de *clústeres* de muertes fetales en Pernambuco, entre 2013 y 2022.

## MÉTODO

### Diseño del Estudio

Se realizó un estudio ecológico que utilizó los municipios de residencia como unidad de análisis.

### Lugar, Población y Criterios de Selección

El estudio se llevó a cabo en el estado de Pernambuco, que tiene una extensión territorial de 98 067,877 km^2^ y alberga una población de 9 058 931 habitantes en 184 municipios distribuidos en 12 regiones sanitarias: I (19 municipios), II (20 municipios), III (22 municipios), IV (32 municipios), V (21 municipios), VI (13 municipios), VII (7 municipios), VIII (7 municipios), IX (11 municipios), X (12 municipios), XI (10 municipios) y XII (10 municipios) (Figura 1)^(13)^.

**Figura 1:** Distribución de los municipios por regiones sanitarias. Pernambuco, 2025.

Elaboración: Autor.

Para la realización de este estudio, se incluyeron todas las muertes fetales de madres residentes en Pernambuco, registradas en el Sistema de Información sobre Mortalidad (SIM) en el periodo comprendido entre 2013 y 2022.

### Recolección de Datos

Se utilizaron datos de muertes fetales registrados en el SIM, alimentados por la declaración de defunción, referentes al periodo estudiado.

Para el cálculo del riesgo relativo (RR), también fue necesario incluir los nacidos vivos de madres residentes en Pernambuco y registrados en el Sistema de Información sobre Nacidos Vivos (Sinasc) en el mismo periodo analizado, alimentado por la declaración de nacidos vivos^(5)^.

Se utilizaron dos periodos de estudio: 2013 a 2017 (primer quinquenio) y 2018 a 2022 (segundo quinquenio). La elección del periodo de análisis se basó en la disponibilidad de la base de datos, cuyo año 2022 era el más reciente disponible para consulta en el momento del análisis de los datos. Se realizó una agregación de los datos por quinquenio para garantizar una mayor estabilidad en los análisis.

Los datos utilizados se consultaron el 20 de diciembre de 2024 y están disponibles en la plataforma electrónica del Departamento de Informática del Sistema Único de Salud (DATASUS) (https://datasus.saude.gov.br/informacoes-de-saude-tabnet/).

### Análisis y Tratamiento de los Datos

Se analizaron variables relacionadas con las características de la madre: edad de la madre en años (<20, 20-34 y >34), escolaridad de la madre en años (<8 y ≥8), tipo de embarazo (único y gemelar o múltiple), semanas de gestación (<37 y ≥37), tipo de parto (vaginal y cesárea). Además de las variables relacionadas con las características del feto: sexo (masculino y femenino), peso al nacer (<2500 g y ≥2500 g) y lugar donde se produjo la muerte (centro de salud y otros lugares).

Se aplicó estadística descriptiva y prueba de Chi-cuadrado con nivel de significación <0,05 para la comparación de proporciones mediante el programa R, versión 3.6., para las muertes fetales agrupadas en quinquenios (2013–2017 y 2018–2022). Mediante el programa de acceso gratuito SatScan 8.2.1, que permite detectar aglomeraciones espaciales, se calculó el riesgo relativo (RR) y se realizó un análisis espacial utilizando la técnica de estadística de barrido espacial. Esta técnica crea ventanas circulares de tamaños variables, cuyo centro se mueve en la superficie del mapa de manera que abarca un conjunto de vecinos cercanos que corresponden a un área geográfica^(14)^. Al moverse por todos los centroides, el radio varía continuamente desde cero hasta el radio máximo, que en este estudio incluyó hasta el 10% de la población total. La función de probabilidad se maximizó en todas las ventanas y se identificó la agrupación más probable. La razón de probabilidad para esta ventana constituyó la prueba estadística de la razón de probabilidad máxima.

El modelo probabilístico adoptado fue del tipo Poisson, en el que, para la confirmación de aglomeraciones espaciales de muertes fetales, se consideró como hipótesis nula que el total de muertes fetales del estado se distribuyen de manera homogénea en todos los municipios, siendo proporcional al total de nacimientos (muertes fetales y nacidos vivos). La hipótesis alternativa fue que el número total de muertes fetales se distribuye de manera heterogénea, lo que da lugar a agrupaciones espaciales de muertes.

La distribución bajo la hipótesis nula y el valor de p simulado correspondiente se obtuvieron repitiendo el mismo ejercicio analítico en un gran número de réplicas aleatorias (999) del conjunto de datos generados bajo la hipótesis nula, en una simulación de tipo Monte Carlo^(14)^. La hipótesis nula se rechazó cuando p < 0,05 para la aglomeración más probable, lo que indica que en esa región se observó que el riesgo de muerte fetal entre los nacidos totales es mayor que el riesgo esperado en la misma región.

### Aspectos Éticos

El estudio no requirió la aprobación de un Comité de Ética en Investigación, de conformidad con la Resolución n.º 674/22 del Consejo Nacional de Salud^(15)^, ya que se utilizaron datos secundarios de dominio público, agregados, que no permiten la identificación individual.

## RESULTADOS

En el período estudiado, se registraron 15 336 muertes fetales, de las cuales 8132 (53%) se produjeron en el primer quinquenio (2013 a 2017) y 7204 (47%) en el segundo (2018 a 2022). La comparación de las características del parto y el nacimiento de las muertes fetales ocurridas en el primer y segundo quinquenio mostró una diferencia estadísticamente significativa para las variables edad de la madre (p < 0,001), nivel educativo de la madre (p < 0,001), tipo de parto (p = 0,001), sexo (p = 0,019) y peso al nacer (p = 0,03) (Tabla 1).

**Tabla 1:** Características de las muertes fetales por quinquenio entre 2013 y 2022 – Pernambuco, Brasil.

Variables (n 15.336)	Óbito fetal		*p-valor*
	2013–2017 n (%)	2018–2022 n (%)	
**Edad de la madre [n = 13.988 (a)]**			**<0,001**
<20	1.557 (57,5)	1.150 (42,5)	
20–34	4.630 (52,8)	4.146 (47,2)	
>34	1.207 (48,2)	1.298 (51,8)	
**Escolaridad de la madre [n = 12.578 (b)]**			**<0,001**
<8	3.458 (59,3)	2.371 (40,7)	
≥8	3.279 (48,6)	3.470 (51,4)	
**Tipo de gravidez [n = 14.746 (c)]**			**0,305**
Única	7.351 (52,9)	6.547 (47,1)	
Doble o más	443 (52,2)	405 (47,8)	
**Semanas de gestación [n = 13.445 (d)]**			**0,082**
<37	5.010 (50,9)	4.824 (49,1)	
≥37	1.906 (52,6)	1.715 (47,4)	
**Tipo de parto [n = 14.616 (e)]**			**0,001**
Vaginal	5.918 (53,6)	5.126 (46,4)	
Cesárea	1.804 (50,5)	1.768 (49,5)	
**Sexo [n = 14.855 (f)]**			**0,020**
Masculino	4.086 (52,1)	3.761 (47,9)	
Femenino	3.783 (54)	3.225 (46)	
**Peso al nacer [n = 14.243 (g)]**			**0,029**
<2.500g	5.333 (52,4)	4.850 (47,6)
≥2.500g	2.209 (54,4)	1.852 (45,6)	
**Óbito en relación al parto [n = 13.309 (h)]**			**<0,001**
Antes del parto	7.352 (53,4)	6.422 (46,6)	
Durante el parto	329 (44,4)	412 (55,6)	
**Lugar donde ocurrió el óbito [n = 15.258 (i)]**			**<0,001**
Establecimiento de salud	7.832 (53,3)	6.856 (46,7)	
Otros	262 (46)	308 (54)	

Número/porcentaje de ignorados

(a) 1348/8,8%

(b) 2758/18%

(c) 590/3,9%

(d) 1881/12,3%

(e) 720/4.7%

(f) 481/3.4%

(g) 1,093/7.1%

(h) 825/5.4%

(i) 78/0.5%

Las características maternas de edad inferior a 20 años (n = 1557; 57,5%) y escolaridad inferior a 8 años (n = 3458; 59,3%) se observaron en mayor número en el primer quinquenio. El parto vaginal se observó en mayor número en el primer quinquenio (n = 5918; 53,6%). En relación con el feto, el sexo masculino (n = 4086; 52,1%) y el peso al nacer inferior a 2500 g (n = 5332; 52,4%) presentaron un mayor número en el primer quinquenio. En cuanto a las características del lugar de la muerte, se observó una diferencia estadísticamente significativa (<0,001), con una mayor incidencia de muertes durante el parto (n = 412; 55,6%) y fuera de los establecimientos de salud en el segundo quinquenio (n = 308; 54%) (Tabla 1).

Se identificaron agrupaciones espaciales en cada uno de los periodos estudiados. En el primer quinquenio (2013–2017), se identificaron tres *clústeres* estadísticamente significativos, formados por 41 municipios del Agreste y el Sertón de Pernambuco, que en conjunto concentraron el 14% del total de nacimientos y abarcaron el 17,9% de las muertes fetales. Destaca el grupo 1, ubicado predominantemente en el Sertón del estado, formado por 15 municipios pertenecientes a las regiones sanitarias V (1 municipio), VI (10) y XI (4), que tuvieron un riesgo de muerte fetal 50% mayor que en los demás municipios (Figura 2, Tabla 2).

**Figura 2:** Aglomeraciones espaciales de riesgo de muerte fetal (n = 15 336), Pernambuco, Brasil, 2013 a 2017 (A); 2018 a 2022 (B).

**Tabla 2:** Características de los conglomerados espaciales de muertes fetales (n = 15 336) - Pernambuco, Brasil, 2013 a 2017; 2018 a 2022.

	Nº de óbitos							
	*Cluster*	Nº de municipios	Observados	Esperados	% Óbitos	% Población	Riesgo Relativo (RR)	p-valor
2013–2017	1	15	549	365	6,8	5,0	1,50	<0,001
	2	16	485	396	6,0	5,0	1,24	0,005
	3	10	409	328	5,1	4,0	1,26	0,006
2018–2022	1	26	829	615	11,6	9,0	1,39	<0,001
	2	12	431	349	6,0	5,0	1,25	0,007
	3	9	190	141	2,6	2,0	1,36	0,04

En el segundo quinquenio (2018–2022), también se identificaron tres grupos estadísticamente significativos, formados por 47 municipios ubicados en el Sertón, el Agreste y la Zona de la Mata de Pernambuco. Estos agregados representan el 15,4% del total de nacimientos del estado y concentran el 20,2% de las muertes fetales (Figura 2, Tabla 2). El grupo 1, formado por 26 municipios ubicados en las regiones sanitarias V (3 municipios), VI (12), VII (2) y XI (9). En este grupo, el riesgo de que los nacimientos totales constituyeran muertes fetales fue un 39% mayor en comparación con otros municipios (Figura 2, Tabla 2).

## DISCUSIÓN

En el periodo estudiado, la mayor parte de las muertes fetales se produjeron en el primer quinquenio. Las características maternas de edad y nivel educativo, además de las características fetales relativas al tipo de parto, sexo, peso al nacer y lugar de la muerte, se relacionaron con el quinquenio analizado. Las muertes fetales de madres menores de 20 años, con bajo nivel educativo, y los nacidos por parto vaginal, de sexo masculino y con bajo peso, se produjeron principalmente en el primer quinquenio. Las muertes ocurridas fuera de los establecimientos de salud presentaron un mayor número en el segundo quinquenio. Las regiones sanitarias V, VI y XI presentaron *clústeres* con alto riesgo de muerte fetal en los dos quinquenios analizados.

En este estudio, se observó un mayor porcentaje de muertes fetales en madres de menor edad, principalmente en el primer quinquenio analizado. Un estudio realizado en Vietnam señala que el embarazo precoz aumenta el riesgo de mortalidad materna, además de contribuir a la existencia de complicaciones obstétricas que pueden provocar la muerte del feto, como consecuencia del crecimiento intrauterino reducido, el parto prematuro y el bajo peso al nacer^(16)^.

El bajo nivel educativo de las madres se observó sobre todo en el primer quinquenio. Una investigación realizada en el municipio de Río de Janeiro observó mayores tasas de mortalidad de los hijos de madres con bajo nivel educativo en comparación con los hijos de madres con alto nivel educativo, especialmente en lo que respecta a la muerte fetal^(17)^.

En este estudio, se observó que la mayoría de los partos fueron vaginales. De manera similar, una investigación realizada en Asia demostró una mayor frecuencia de muertes fetales nacidos por parto vaginal^(1)^. La literatura recomienda esta vía de parto cuando hay buenas condiciones de supervivencia materna y fetal, ya que reduce el riesgo de prematuridad, complicaciones placentarias y ruptura uterina en futuros embarazos^(18)^.

Sin embargo, en emergencias debidas a complicaciones obstétricas, la elección del parto vaginal debe evaluarse, ya que puede poner en peligro la supervivencia de la madre y el bebé^(19)^. Según se observó en un estudio de casos y controles, la cesárea,en comparación con el parto vaginal, puede reducir a la mitad las posibilidades de muerte fetal^(1)^.

La mayoría de las muertes se produjeron entre los fetos de sexo masculino. Una investigación realizada en el municipio de São Paulo también constató que la mortalidad fetal afecta principalmente al sexo masculino^(20)^. Este resultado puede estar relacionado con el hecho de que el desarrollo pulmonar es más lento en los individuos de sexo masculino en comparación con los de sexo femenino. La maduración pulmonar tardía conlleva una mayor susceptibilidad a las infecciones respiratorias que provocan la muerte^(21)^.

La mortalidad fue mayor entre los fetos con bajo peso al nacer. La literatura señala que, a medida que disminuye el peso al nacer, aumenta el riesgo de mortalidad fetal^(20)^. Para reducir los nacimientos con peso inadecuado, es fundamental el acceso oportuno a la atención prenatal durante el primer trimestre de gestación, con una frecuencia de seis o más consultas y una asistencia de calidad^(22)^. Durante la atención prenatal, la oferta de vacunación, la realización de exámenes de laboratorio, el diagnóstico oportuno y el tratamiento adecuado pueden contribuir a la disminución de la mortalidad fetal^(23)^.

En cuanto al lugar donde se produjo el óbito, en el presente estudio se observaron muertes fetales que ocurrieron fuera de los establecimientos de salud, principalmente en el segundo quinquenio analizado. Según una investigación realizada en Bangladesh, el acceso oportuno a los servicios de asistencia al parto puede reducir a la mitad la mortalidad fetal y neonatal temprana^(24)^. Esto se debe a que el desplazamiento en el momento del parto contribuye a la ocurrencia de muertes, ocasionadas por la desigualdad en el acceso a los servicios de salud obstétrica, que afectan sobre todo a las embarazadas con mayor vulnerabilidad socioeconómica, según una investigación nacional^(2)^.

Al investigar las muertes fetales ocurridas fuera de los establecimientos de salud en el segundo quinquenio de este estudio (2018–2022), es importante considerar la pandemia de COVID-19, decretada en el año 2020^(25)^. Un estudio realizado en la India señala que la demora en la llegada de la embarazada a los servicios de salud, la realización tardía de procedimientos quirúrgicos y el peregrinaje para dar a luz aumentaron el riesgo de muerte fetal durante el período pandémico^(26)^. Es sabido que durante la pandemia, para dar prioridad a la atención de los casos de COVID-19, se interrumpieron los servicios presenciales de atención prenatal y se redujo la atención obstétrica, lo que puede haber contribuido a la ocurrencia de muertes fetales^(26)^.

Los resultados del análisis de barrido espacial permitieron identificar, en los dos quinquenios analizados, *clústeres* de mayor riesgo de mortalidad fetal en las regiones sanitarias V, VI y XI. Para el segundo quinquenio, se observó la aparición de un grupo en la región VII. Además de presentar una alta mortalidad fetal, los municipios de las regiones V, VI y XI también se consideran áreas prioritarias para el desarrollo de acciones destinadas a reducir las carencias sociales, de acuerdo con un trabajo anterior realizado^(27)^.

La presencia de grupos de mortalidad fetal en algunas regiones también puede indicar debilidades en la Red Materno-Infantil de Pernambuco/Red Cegonha (nombre del Programa de nacimientos seguros), que tiene como objetivo garantizar a la mujer el derecho a la planificación reproductiva y a la atención humanizada durante el embarazo, el parto y el puerperio, así como al niño el derecho a un nacimiento seguro. Entre las deficiencias observadas en la red, se constata que en los municipios de la V región sanitaria (RS) no es posible identificar una maternidad de referencia para embarazos de alto riesgo. En las regiones VI y XI no hay camas para embarazos de alto riesgo ni camas en la unidad de terapia intensiva (UTI) neonatal. La maternidad de referencia para embarazos de alto riesgo ubicada en la RS VII, a pesar de contar con camas para embarazos de alto riesgo, no tiene UTI neonatal^(28)^.

De manera similar al presente estudio, el análisis espacial se ha utilizado en la literatura para señalar la distribución de las muertes fetales en los territorios, como se observa en una investigación realizada en Uganda^(29)^. El análisis de datos geor-referenciados también puede contribuir a la comprensión de los contextos locales y los factores asociados a la ocurrencia de las muertes, como se puede observar en un estudio realizado en Mozambique^(30)^. Además, permite identificar las áreas que deben ser prioritarias en la toma de decisiones que contribuyan a la reducción de la tasa de mortalidad fetal. Mediante el análisis espacial, es posible monitorear la eficacia de las políticas públicas, lo que puede identificarse en una investigación que analizó la calidad de los servicios de atención primaria y la reducción de la mortalidad infantil en Brasil^(12)^.

Así, se refuerza la importancia de elaborar nuevas investigaciones que verifiquen el riesgo de mortalidad en diferentes territorios, identifiquen los factores asociados a la distribución espacial de la mortalidad, señalen las áreas prioritarias para las intervenciones en salud y monitoreen las acciones realizadas para mejorar los indicadores de salud. Las limitaciones de este estudio pueden estar relacionadas con el uso de datos secundarios, la posible subnotificación de muertes fetales y la incompletitud de los sistemas de información en salud. Lo que puede dar lugar a una subestimación de los riesgos calculados. Además, la unidad de análisis puede ocultar desigualdades intramunicipales.

Los resultados de este estudio pueden contribuir a la enfermería en la identificación de áreas con mayor riesgo de muerte fetal. Al discutir este problema de salud pública y proporcionar información para la toma de decisiones, esta investigación puede orientar la asignación de recursos para fortalecer las prácticas de atención que permitan reducir las muertes evitables.

## CONCLUSIÓN

La caracterización de las muertes fetales presentada en los resultados de este estudio contribuye a la comprensión de las circunstancias que implicaron la ocurrencia de las muertes. Las variables estadísticamente significativas en los quinquenios analizados fueron: edad de la madre, nivel educativo de la madre, tipo de parto, sexo, peso al nacer, muerte en relación con el parto y lugar de ocurrencia de la muerte. El análisis espacial verificó *clústeres* de mayor riesgo de muerte fetal en las regiones V, VI, VII y XI. Este análisis también permitió indicar áreas que necesitan intervención de planificación en salud con acciones inmediatas destinadas a mejorar la red de asistencia materna e infantil.

## Data Availability

Todo el conjunto de datos que apoya los resultados de este estudio fue publicado en el propio artículo.
